# A whole family-based physical activity promotion intervention: findings from the families reporting every step to health (FRESH) pilot randomised controlled trial

**DOI:** 10.1186/s12966-020-01025-3

**Published:** 2020-09-22

**Authors:** Justin M. Guagliano, Sofie M. Armitage, Helen Elizabeth Brown, Emma Coombes, Francesco Fusco, Claire Hughes, Andrew P. Jones, Katie L. Morton, Esther M. F. van Sluijs

**Affiliations:** 1 MRC Epidemiology Unit and Centre for Diet and Activity Research, University of Cambridge, Cambridge, UK; 2 Norwich Medical School and Centre for Diet and Activity Research, University of East Anglia, Norwich, UK; 3 Department of Public Health and Primary Care, University of Cambridge, Cambridge, UK; 4 Centre for Family Research, University of Cambridge, Cambridge, UK

**Keywords:** Youth, Parent, Mothers, Fathers, Mums, Dads, Co-participation, Co-physical activity

## Abstract

**Introduction:**

This study assessed the feasibility and acceptability of FRESH (Families Reporting Every Step to Health), a theory-based child-led family physical activity (PA) intervention delivered online. We also assessed the preliminary effectiveness of the intervention on outcomes of interest and whether pre-specified criteria were met to progress to a full-scale definitive trial.

**Methods:**

In a three-armed randomised pilot trial, 41 families (with a 7–11-year-old index child) were allocated to a: ‘family’ (FAM), ‘pedometer-only’ (PED), or a no-treatment control (CON) arm. The FAM arm received access to the FRESH website, allowing participants to select step challenges to ‘travel’ to target cities around the world, log their steps, and track progress as families virtually globetrot. FAM and PED arms also received family sets of pedometers. All family members could participate in the evaluation. Physical (e.g., fitness, blood pressure), psychosocial (e.g., social support), behavioural (e.g., objectively-measured PA), and economic (e.g., expenditure for PA) data were collected at baseline, 8- and 52-weeks.

**Results:**

At 8- and 52-weeks, 98 and 88% of families were retained, respectively. Most children liked participating in the study (> 90%) and thought it was fun (> 80%). Compared to the PED (45%) and CON (39%) arms, a higher percentage of children in the FAM (81%) arm reported doing more activities with their family. Adults agreed that FRESH encouraged their family do more PA and made their family more aware of the amount of PA they do. No notable between-group differences were found for childrens’ minutes in moderate-to-vigorous PA. Sizeable changes of 9.4 (95%CI: 0.4, 18.4) and 15.3 (95%CI: 6.0, 24.5) minutes in moderate-to-vigorous PA was found for adults in the FAM group compared to those in the PED or CON groups, respectively. No other notable differences were found.

**Conclusion:**

This study demonstrates feasibility and acceptability of the FRESH intervention. All progression criteria were at least partially satisfied. However, we failed to recruit the target sample size and did not find a signal of effectiveness on PA particularly long-term or in children. Further refinements are required to progress to a full-scale trial.

**Trial registration:**

This study was prospectively registered (ISRCTN12789422) on 16/03/2016.

## Introduction

The direct healthcare costs of physical inactivity in the United Kingdom (UK) is among the highest in Europe and is estimated to be INT$1.5 billion [1]. Approximately one-third of adults in the UK are insufficiently physically active, falling short of achieving the national recommendation of at least 150 min of moderate- or 75 min of vigorous-intensity physical activity weekly [2, 3]. Adults with school-aged children are particularly at risk for physical inactivity [4, 5], and a recent review showed that young adults exhibited greater declines in physical activity over the transition to parenthood compared to those without dependent children [6].

Half of UK children fail to meet the national recommendation of 60 min of daily moderate-to-vigorous intensity physical activity (MVPA) [7]. Observational data also reveal that children are less active after school and on weekends than during school time, and that activity levels decline most steeply outside of school [8–10]. Targeting children and adults as a family therefore appears to be a promising avenue for promoting physical activity [11, 12].

Previous research suggests that involving family members is critical for sustained behaviour change [13–15] and home-based family physical activity interventions are potentially more effective than those requiring the family to travel to community or other intervention locations [16, 17]. Many studies, however, centre around promoting child physical activity instead of considering the family as a unit that may work together to change behaviour [18]. Our recent feasibility study [19] evaluated an intervention that specifically targeted whole family engagement. The findings showed that it was feasible to deliver and evaluate a family-targeted physical activity promotion intervention with high acceptability from participating families.

Building upon this work, here we present the findings from the Families Reporting Every Step to Health (FRESH) pilot trial. The primary aim of this pilot trial was to assess the feasibility and acceptability of the revised recruitment strategy, intervention, and outcome evaluation (i.e., after feasibility testing [19]). Secondary aims were: (1) to explore the preliminary effectiveness of the intervention on potential outcomes of interest and; (2) to assess whether pre-specified criteria were sufficiently met to warrant progression to a full-scale definitive trial.

## Methods

A detailed description of the study protocol has been published elsewhere [20]; a brief summary of the methods is provided below. We received ethical approval from the Ethics Committee for the School of the Humanities and Social Sciences at the University of Cambridge (ID number: 17/113) and this study was prospectively registered (ISRCTN12789422).

### Study overview

This pilot trial was a three-armed, parallel-group, randomised controlled pilot trial using a 1:1:1 allocation ratio and included follow-up assessments at 8- and 52-weeks post-baseline. After baseline assessments, families were randomly allocated to one of the three study arms: (1) family arm, (2) pedometer-only arm, or (3) no-intervention control arm. Families were recruited from the counties of Norfolk and Suffolk, UK.

Recruitment difficulties led to an 8-week extension of the originally planned 16-week period. At minimum, families with at least one child in school Years 3–6 (aged 7–11 years, hereafter referred to as the index child) were eligible to participate if at least one adult responsible for the index child and living in the main household (hereafter referred to as the index parent) provided consent. However, we ideally sought to recruit whole families, that is, all adults and children living in the main household with the index child. If requested, we also enabled the inclusion of parents or siblings that lived outside the main household or extended family members (e.g., grandparents) living inside or outside the index child’s main household. All participants were required to be able to perform light-intensity physical activity, have access to the Internet, and have sufficient understanding of the English language to provide informed consent. For this study, we permitted family members to take part in the intervention irrespective of their participation in the accompanying evaluation and vice versa.

We aimed to recruit 60 families using a multi-faceted recruitment strategy that was informed by our prior work [21, 22]. This approach targeted adults and children, included a wide range of physical settings (such as schools, employers, community settings including community centres, shopping centres, GP surgeries), used electronic media (e.g., social media, television news). It was also based on direct (e.g., face-to-face recruitment during school pick up) and indirect recruitment strategies (e.g., posting recruitment material on parent groups on social media platforms). Following dissemination of recruitment materials, families were encouraged to express interest in participating to the study team, who conducted a screening assessment and scheduled a baseline appointment with eligible families. Prior to baseline assessments, written informed consent was obtained for all participating adults, alongside written parental consent and child assent for each participating child. After baseline assessments, families were randomised in blocks of six and stratified by county (i.e., Norfolk or Suffolk) by an independent statistician using a computer-generated algorithm.

### Intervention protocol

#### Family arm (FAM)

The development, feasibility, acceptability, and refinements made to the intervention prior to the current pilot trial have been previously described [19, 20], including a detailed description of the FRESH intervention as implemented in the pilot [20]. In summary, families in the FAM arm received a theory-based intervention that was delivered online and aimed at increasing physical activity for the whole family [23–25]. Intervention participation started with a 1-h kick-off meeting in which a member of the research team introduced families to the intervention website, distributed pedometers to all family members, and prompted the first of weekly ‘family time’ meetings. The index child or children (if multiple) were designated the role of team captain(s) and they led weekly ‘family time’ meetings. During these meetings, families completed family action planners and accessed the FRESH website which enabled them to choose weekly step challenges. Family action planners prompted families to plan weekly family physical activities to assist in meeting their step challenge for a given week. It was intended that families would plan activities they would do together as a family; however, participants had the flexibility to also set individual level goals. The action planners also prompt families to monitor weekly step counts, discuss any potential upcoming barriers for physical activity and strategies to overcome them. Index children will be allocated as their family’s ‘team captain’ leading in challenge selection and uploading steps on the FRESH website. Families retained their pedometers and were permitted to use the website for as long as they liked, with continuing support.

#### Pedometer-only arm (PED)

Following baseline, families allocated to the PED arm were mailed pedometers for all family members and generic family physical activity promotion information produced by Walk4Life, a sub-brand of Change4Life (www.nhs.uk/change4life). Example information provided included tips to get walking daily and games that can be played while walking. Like FAM families, they continued to receive generic information fortnightly on four occasions.

#### Control arm (CON)

CON families were asked to carry on as normal and did not receive access to the intervention website, pedometers, or any generic information.

### Outcome evaluation measures

All consenting family members were assessed at baseline, 8, and 52 weeks post-baseline and data were collected in the family home by two trained research staff.

#### Accelerometer and GPS assessment

Participants were asked to simultaneously wear an ActiGraph GT3X+ tri-axial accelerometer (ActiGraph LLC; Pensacola, Florida) and QStarz Travel Recorder BT1000X global positioning system (GPS) monitor (QStarz; Taipei, Taiwan) on each hip during waking hours for 7 consecutive days. After the 7 days of wear, participants either posted the devices back to the research team using pre-paid envelopes or the research team picked up the devices at an agreed time.

The accelerometer was initialised to record step counts and acceleration using a sampling frequency of 50 Hz. Data from the device were then downloaded and interpolated to a 10 s epoch using the ActiLife software. A valid week for the accelerometery was defined as a minimum of 480 min/day from 3 days (including 1 weekend day) over the 7-day measurement period. Non-wear was defined as ≥90 mins of consecutive zeros [26]. The cut points of Evenson et al. [27] and Troiano et al. [28] were used to estimate physical activity for children and adults, respectively.

The GPS device recorded participants’ locations at a 10 s interval with an accuracy of approximately 3 m. Data from the GPS devices were downloaded and entered into the ArcGIS v10.3 (ESRI Inc., California, USA) Geographical Information System, and then longitude and latitude values were converted to easting and northing values respectively according to the British National Grid coordinate reference system [29].

The accelerometer and GPS data were then integrated based on their date and time-stamps using bespoke software written in Java. From the integrated accelerometer and GPS data, individual measures of time spent with and without other family members present were computed. This was undertaken using a script written in STATA v16 (StataCorp LLC, Texas, USA) that calculated the straight-line distance between each participant and every member of their family for all 10 s intervals, based on each participant’s easting and northing locations. To identify physical activity undertaken together, a distance of ≤50 m was taken as being indicative of the same location of members of the family during any given 10 s interval. This distance was selected because it is approximately equivalent to a ball court (e.g. tennis, basketball) or a large residential garden [30].

#### Physical health outcomes

Aerobic fitness (via predicted VO_2 max_) was measured using an 8-min submaximal step test (with 2-min rest) on all participants ≥7 years [31]. Height and weight were measured with a portable stadiometer and digital scale, respectively. Waist circumference was measured twice, using a non-elastic tape measure (third measure taken if the first two differed by ≥3 cm). Body mass index was calculated, and converted into age- and sex-specific percentiles using standard growth charts for children [32].

#### Behavioural and psychosocial measures

Behavioural and psychosocial measures were measured via questionnaires for participants ≥4 years. Measures included: screen-time use [33–36]; quality of life [37–40]; family co-participation in physical activity [36]; physical activity awareness [41, 42]; family social norms for physical activity [43, 44]; family support [43]; motivation for physical activity [45, 46]; and children’s perceived autonomy, competence, and relatedness [46].

#### Family functioning

The Fictional Family Holiday Paradigm was used to assess family functioning via family relationships [47] and connectedness [48]. In this observational paradigm, each family was asked to spend 10 min planning and discussing a fictional week-long holiday itinerary with unlimited budget. The video-recorded activity was then transcribed and coded by trained research assistants per time point for: ‘power sharing’ (i.e., taking turns speaking); positive talk (e.g., expressions of amity, elicitation of family members’ viewpoints, agreement, compromise) [48], and discussions that revolve around physical activity.

#### Family out-of-pocket expenditure for physical activity

Physical activity related expenditure for each family member was collected via questionnaire. The questionnaire comprised two questions about expenditure related to membership fees and subscriptions (e.g., for sports clubs, fitness centres) and sports equipment (e.g., sportswear, gadgets) and was completed by the same adult at each time point for their whole family.

### Feasibility and acceptability assessment

A mixed-methods process evaluation was conducted at 8 weeks post-baseline. Adults responded to open-ended and Likert-scale questions (4-point; 1 = strongly disagree, 4 = strongly agree) and children responded to dichotomous ‘yes/no’ questions regarding their overall opinion of FRESH, the intervention components, measurements, and suggestions for improvement. In addition, semi-structured focus groups were conducted with willing families (*n* = 5 FAM; *n* = 4 PED; *n* = 1 CON). This focused on families’ experience taking part in the trial, perceived acceptability of individual intervention components, intervention fidelity, challenges/barriers encountered, and suggested improvements, as appropriate based on study arm allocation. All focus groups were transcribed verbatim. We also explored FAM arm families’ engagement with the intervention website through Google Analytics (e.g., page views, challenges accepted/completed) and assessed aspects of the recruitment process (e.g., recruitment duration, resources used, comparisons of recruitment strategies). Lastly, intervention costs were also calculated.

### Progression criteria assessment

Table 1 outlines pre-specified criteria used to inform progression to a definitive trial. Where applicable, quantitative and qualitative findings were taken into account to assess whether a criterion was met.

**Table 1:** Descriptions and assessments of pre-specified criteria used to inform progression to a definitive trial

Description	Criterion met?	Assessment
1. > 75% of families upload steps at least 6 times in the first 3 months of pilot study.	Yes	• 86% of families uploaded steps > 6 times in first 3 months (mean ± SD = 11 ± 4 uploads).
2. Demonstrable feasibility of recruiting 20 families/month (accounting for increased staffing in future definitive trial) and retaining 75% of index children at 52-week follow up.	Partially	• The average recruitment rate was approximately ~ 7 ± 5 families/month (range = 2–15 families/month). • 88% of index children were retained at 52-week follow up.
3. Intervention optimisation feasible (identified adaptations are practical, affordable, acceptable).	Yes	• Focus groups revealed few suggested changes to the website; however, some families indicated a preference for the intervention to be delivered through a mobile phone app rather than a website. • Many families suggested receiving wrist-worn pedometers.
4. Evidence to suggest an adequately powered trial would require a feasible number of participants (*N* = 250 is considered logistically feasible and providing sufficient power).	Yes	• Post-hoc sample size calculations were performed and to provide 80% power to detect a difference of 10 mins in MVPA in index children, 27 index children/family are needed, using a standard deviation of 16.3 mins of MVPA and a pre-post correlation of 0.63. ○ With 3 arms: 27 index children * 4 people/family * 3 arms; *N* = 81 families (~ 324 total participants) ○ With 2 arms: 27 index children * 4 people/family * 2 arms; *N* = 54 families (~ 216 total participants) • Therefore, to conduct an adequately powered trial with a feasible number of participants it will have to be a 2-armed study.
5. Discontinuation of trial arm based on evidence of harm or limited acceptability/feasibility.	Yes	• There were no reports of harm, however, during the focus groups some pedometer only-armed families unknowingly indicated that they would have liked to receive several elements that were delivered to families in the family-arm (e.g., step challenges). This finding provides some evidence to suggest the pedometer only-arm could be discontinued in future.

### Data analysis

#### Quantitative data

Statistical analyses of the primary and selected secondary outcomes were conducted using analysis of covariance (adjusting for baseline values) in Stata (version 15; StataCorp. TX: StataCorp LP), stratified by age group (adults vs children). Participants with missing values at baseline were included in the analysis using the missing indicator method [49]. An estimate of effect and 95% confidence interval were calculated for primary and selected secondary outcomes; no *p*-values were calculated. We stratified analyses by index of multiple deprivation (IMD) score (high/low IMD determined by median split) and sex to explore signals of subgroup effects in all outcomes.

To inform one of the progression criteria, post-hoc sample size calculations were calculated to provide 80% power to detect a difference of 10 mins in MVPA in index children (*p* < 0.05), using a standard deviation of 16.3 mins of MVPA and a pre-post correlation of 0.63 (values obtained from 52-week follow-up).

#### Economic analyses

The intervention costs were calculated by using a micro-costing approach [50]. Table 2 reports the resources used per family, and their monetary value, alongside the subsequent cost per item. All families were assumed to incur the same intervention cost, except from the pedometers, which was based on the number of participants per family. The reported family physical activity expenditure was summed per each family and the mean costs per family was calculated at each time point.

**Table 2:** Intervention cost components and cost per item/family

Items	Resource use	Unit cost (£)	Cost/item (£)
**Family arm**			
Kick-off meeting	75 min^+^	0.33	25
Accompanying booklet	12 pages	0.20	2.4
Pedometers	1 pedometer per study participant	4.00	4
Personalised messages	118 Minutes^++^	0.33	39.3
Online and tangible rewards	5 cards	0.20	1
**Total cost**	**–**	**–**	**71.7**
**Pedometer-only arm**			
Pedometers	1 pedometer per study participant	4.00	4
Accompanying booklet	12 pages	0.20	2.4
**Total cost**	**–**	**–**	**6.4**

**Note.** ^+^ Kick-off meeting duration: 60 min; travelling time:15 min. ^++^ The personalised messages were posted for 11 weeks, which required on average 10.72 min per week per family

We conducted a comparative analysis based on the complete-cases dataset at 52 weeks. A linear regression was used to estimate the between-groups differences in mean costs per family, accounting for the cost at baseline (incurred during the 3 months prior to baseline) [51]. The 95% CIs were constructed by resampling the dataset 5000 times performing a non-parametric bootstrap with replacement.

#### Qualitative data

A content analysis was conducted using existing guidelines [52] to explore the feasibility and acceptability of the revised FRESH intervention, outcome evaluation, and suggestions for further intervention optimisation via family focus groups.

## Results

### Recruitment and retention

**Fig. 1:**
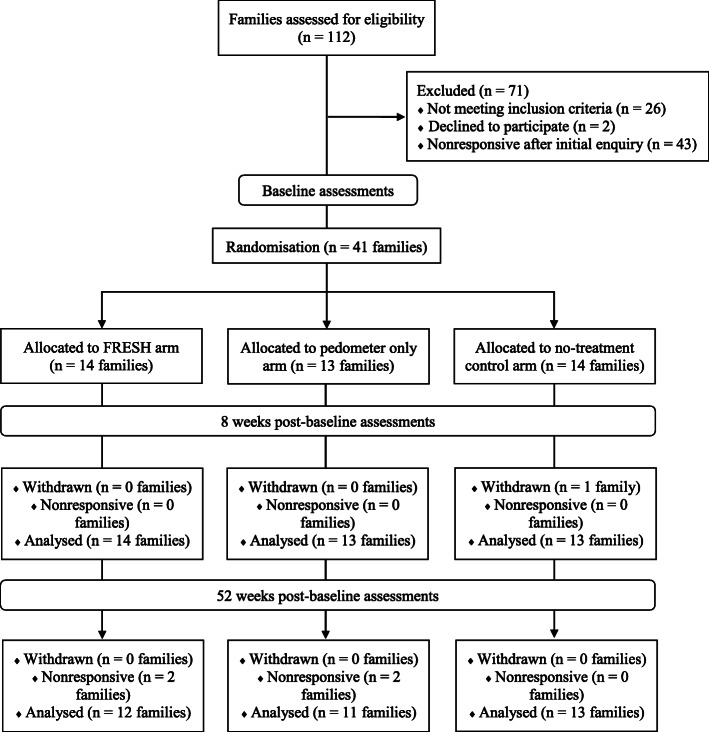
Participant flow diagram

Table 3 provides a summary of recruitment sources used in this study and Fig. 1 shows the recruitment flow. Expressions of interest occurred at a rate of 4–5 families/week over the 24-week recruitment period. Approximately 77% of families expressing interest were eligible for participation and 48% of eligible families were enrolled, with an enrolment rate of ~ 1–2 families/week.

**Table 3:** Recruitment sources

	Schools	Employers	Community^**a**^	Traditional media^**b**^	Social media^**c**^	Referral	Unknown	Total
Approached	87	102	56	N/A	12	N/A	N/A	257
Agreed	16	10	7	N/A	5	N/A	N/A	38
Families reached	~ 1641	~ 8761	~ 1740	~ 2371	24,333	N/A	N/A	~ 38,846
Expressions of interest	42	11	26	22	1	4	6	112
Eligible	41	9	22	7	1	4	2	86
Families enrolled	23	7	4	4	0	3	0	41

**Note.** ^a^Included settings such as: parkrun, community centres, swimming pools, Scouts/Cubs/Guides, shopping centres, local community events. ^b^Included a story highlighting the study on a local television news program. ^**c**^Included parent websites or groups on FaceBook and Twitter

Of the 41 families enrolled, 73% included all family members (*n* = 30 families). The remaining families either only included index parent-child dyads (*n* = 4 families) or the index parent, index child, and an additional parent and/or children (*n* = 7 families); however, in all cases there were additional adults and/or children living with the index child that chose not to participate in the study. We did not recruit any extended family members or any family members that lived outside the index child’s main household. Consent was obtained for 149 participants, averaging ~ 4 members/family (range = 2–6 family members) and included 39 mothers (95%), 31 fathers (76%), and 41 siblings (from 32 families with an eligible sibling, 78%). Eleven siblings were younger than index children (< 7 years of age), 15 were in the same age category (7–11 years) and 15 were older (> 11 years). Table 4 describes the participant characteristics at baseline. Notably, children in the family arm were older compared to children in the other two arms (FAM: 10.1 ± 2.8y; PED: 8.6 ± 1.9y; CON 8.9 ± 2.7y) and there were fewer girls allocated to the pedometer arm (FAM: 50.0%; PED: 17.4%; CON 48.3). Approximately, 92% of adults reported being married or living as married, 94% of adults reported their ethnicity as white, and the mean ± SD age that adults finished full-time education was 20.5 ± 3.5 years.

**Table 4:** Individual Participant Characteristics at Baseline

	Overall		Family		Pedometer		Control	
	Adults (*n* = 67)	Children (*n* = 82)	Adults (*n* = 21)	Children (n = 30)	Adults (*n* = 24)	Children (*n* = 23)	Adults (*n* = 22)	Children (*n* = 29)
Sex (% female)	56.7	40.2	54.2	50.0	50.3	17.4	57.1	48.3
Age, yr (± SD)	41.3 ± 5.8	9.3 ± 2.6	42.7 ± 5.3	10.1 ± 2.8	39.0 ± 6.2	8.6 ± 1.9	42.2	8.9 ± 2.7
Height, cm (± SD)	171.8 ± 9.1	136.3 ± 15.6	172.4 ± 8.8	140.4 ± 14.8	172.8 ± 9.6	135.1 ± 11.7	170.0 ± 9.0	132.9 ± 18.4
Weight, kg (± SD)	78.1 ± 14.2	32.5 ± 9.6	81.3 ± 13.8	35.2 ± 9.1	76.5 ± 13.0	31.4 ± 7.8	76.3 ± 15.7	30.7 ± 10.9
Body mass index, kg^.^m^− 2^ (± SD)	26.5 ± 4.6	17.1 ± 2.4	27.5 ± 5.0	17.6 ± 2.4	25.6 ± 3.4	16.9 ± 2.3	26.3 ± 5.3	16.8 ± 2.5
Body mass index *Z*-score (± SD)	N/A	0.1 ± 1.1	N/A	0.2 ± 1.1	N/A	0.3 ± 1.1	N/A	0.0 ± 1.1
Waist circumference, cm (± SD)	89.1 ± 12.2	61.0 ± 8.0	93.4 ± 12.0	62.4 ± 9.1	86.6 ± 12.5	62.0 ± 5.8	86.9 ± 11.3	59.0 ± 8.2

At 8- and 52-weeks assessments, 98 and 88% of families were retained (family drop out: *n* = 2 FAM; n = 2 PED; *n* = 1 CON), respectively. Participant loss to follow-up at 52 weeks included 9 adults (*n* = 4 FAM; *n* = 3 PED; n = 2 CON) and 11 children (*n* = 4 FAM; *n* = 3 PED; *n* = 4 CON).

### Intervention feasibility, acceptability, fidelity, and optimisation

Most children reported that they liked taking part in the study (> 90%) and thought it was fun (> 80%). Compared to the PED (45%) and CON (39%) arms, a higher percentage of children in the FAM (81%) arm reported doing more activities with their family at 8-week follow up. Table 5a shows adults’ overall perceptions of FRESH. Scores were generally positive and favoured the FAM over the PED arm. In particular, adults agreed that FRESH encouraged their family do more physical activity and made their family more aware of the amount of physical activity they do. Focus group findings also related to family physical activity, physical activity awareness, and parental modelling, for example:

“It was really fun, it pushed us to get our steps in and do more activities and sports together, you know. I never really thought about how many steps or exercise I’ve done to be honest, so since these [step] counters, I just look and go ‘3,000 [steps] only? I have to do something’. So sometimes they [her 3 sons] come home and they see me… dancing, doing something, or skipping, they say, ‘what are you doing, mum?’ [*laughs*] and I say, ‘I’m just putting effort in to get my steps’ and then they join me, you know. It just made your more aware… I even started walking for small shopping instead of driving just to get my steps up [*laughs*]… small things, you know, it just made you aware.” (Mother of 3, FAM group).

Overall acceptability of the pedometers was fairly high for adults in both the FAM and PED arms (Table 5b). Families in both groups reported that it became habitual to wear the pedometers; one parent stated: “I think it’s become quite habitual now, we pick them up first thing in the morning and take them off last thing at night and they [her children] were quite happy to do that, so that was good from a parent point of view.” (Mother of 2, FAM group). A greater percentage of PED children self-reported that they liked wearing their pedometer compared to FAM children (86% vs. 62%). Also, compared to our previous feasibility study, families’ preference to wear wrist-worn pedometers was emphasised more strongly during focus group discussions in this study.

**Table 5:** Summary Process Evaluation Findings for Adult Participants Assessing the Acceptability of the Families Reporting Every Step to Health (FRESH) intervention

	Family arm (n = 25 adults)	Pedometer-only arm (n = 21 adults)
a) The FRESH study…		
…was fun for my family and I.	3.2 ± 0.7	3.1 ± 0.5
…encouraged my family and I to do more physical activity.	3.2 ± 0.7	2.7 ± 0.8
…has led my family and I to do more physical activity than we did before FRESH.	3.0 ± 0.8	2.4 ± 0.8
…has led my family and I to do more activities (other than physical activity) together than we did before FRESH.	2.6 ± 0.8	2.2 ± 0.5
…has made my family and I more aware of the amount of physical activity we do.	3.6 ± 0.6	3.2 ± 0.7
…is something my family and I would like to continue to be part of.	3.3 ± 0.9	3.2 ± 0.6
b) Regarding the step counter we gave out to log your steps, to what extent do you agree or disagree with the following:		
I didn’t mind wearing it.	3.4 ± 1.0	3.1 ± 1.1
My child/children didn’t mind wearing it.	3.2 ± 1.0	3.2 ± 0.9
It was easy to use.	3.1 ± 0.9	3.6 ± 0.5
I thought it was reasonably reliable at counting steps.	2.8 ± 1.0	3.0 ± 0.6
I used the memory feature to go back and look at the number steps my family and/or I took.	3.0 ± 1.1	2.0 ± 1.1
c) Regarding ‘family time’, to what extent do you agree or disagree with the following:		
It was easy to schedule ‘family time’.	3.0 ± 1.0	N/A
My family consistently scheduled ‘family time’.	2.4 ± 1.0	N/A
My child reminded us about ‘family time’.	3.0 ± 0.9	N/A
My child led/initiated ‘family time’.	3.1 ± 0.8	N/A
d) Regarding the FRESH website, to what extent do you agree or disagree with the following:		
It was easy to use.	3.8 ± 0.7	N/A
I enjoyed using it.	3.4 ± 0.8	N/A
My child/children enjoyed using it.	3.4 ± 0.8	N/A
I thought the website was appealing.	3.5 ± 0.7	N/A
I liked that there were varying degrees of difficulty with the challenges.	3.5 ± 0.8	N/A
I enjoyed the information about the cities.	3.5 ± 0.8	N/A
My child/children enjoyed the information about the cities.	3.4 ± 0.8	N/A
The step converter was useful (e.g., converting swimming to steps).	3.6 ± 0.8	N/A
The resources page was useful.	3.5 ± 1.0	N/A
I enjoyed the recipes.	3.6 ± 1.4	N/A
My child/children enjoyed the recipes.	3.6 ± 1.4	N/A
Logging our steps was easy.	3.5 ± 0.9	N/A

Note. Participants responded on a 4-point Likert scale for each question (1 = strongly disagree; 4 = strongly agree)

Based on a 4-point Likert scale (1 = strongly disagree, 4 = strongly), FAM adults found the kick-off meeting useful (mean ± SD = 3.6 ± 1.0) to help them get started, felt they had enough technical support if needed (3.9 ± 0.6), and found it feasible to schedule ‘family time’ but not consistently so (see Table 5c). Focus groups revealed that families were rarely using their action planners. One parent described: “we probably didn’t fill that [action planner] in as much as we should’ve… we use that [action planner] more to actually record our steps.” (Father of 2, FAM group).

The majority of FAM children found the website easy to use (93%), wanted to keep using it (81%), enjoyed being their family’s team captain (70%), and did not find it too difficult to reach their step goals (65%). Overall, adults’ mean scores were generally positive in relation to the intervention website (see Table 5d). In particular, adults strongly agreed that the website was easy to use and found various website elements to be useful (e.g., the step converter). Parents agreed that their child enjoyed receiving rewards and competence reinforcement after each challenge week (mean ± SD = 3.5 ± 1.2), based on a 4-point Likert scale (1 = strongly disagree, 4 = strongly). When asked in focus groups about suggestions for improvement, PED families suggested elements that were delivered to the FAM group, for example:

“I think if you can walk so many steps and it gets you to a place, like a country or something like that. So maybe there could be mini challenges like you walk to London or walk to Paris, you know, or something. Yeah, something like that would be probably quite good for you guys [referring to her children]. […] We haven’t been around the world, but we’d like to go around the world. […] I think that’s something you can add to this [study]. (Mother of 2, PED group).

Google Analytics data indicated that 59 users accessed the website (~ 4 users/family) with a median (interquartile range) of 2 (1–5) sessions/user, viewing about 5 (2–11) pages/session, for about 7 (3–12) minutes/session. The most common behaviour flow was to log on, access the challenge page (to select a new challenge) and then access the steps page (to add steps to complete their challenge). Families selected an average of 11 challenges and completed 9 of those.

### Findings related to feasibility and acceptability of the outcome evaluation

Data collection took an average of 119.5 ± 26.4 min/family at baseline and 95.0 ± 16.7 and 82.3 ± 35.8 min/family at 8- and 52-week follow up, respectively. Overall, adults disagreed that there were too many measures (mean ± SD = 1.5 ± 0.7) and that data collection took too long (mean ± SD = 1.7 ± 0.8), based on a 4-point Likert scale (1 = strongly disagree, 4 = strongly). Focus group families highlighted the convenience of home-based data collection and, in some cases, it was essential for their participation. One parent indicated: “…it was a lot more convenient you coming to us and you guys being quite flexible in offering us multiple dates and times you could come… if you hadn’t come to us, we probably wouldn’t have participated.” (Father of 1, FAM group). Also, > 80% children reported that they ‘liked’ the measurement sessions. At each time point, > 90% of eligible adults and children completed all measures, except for the submaximal step test (86%) and the video-recorded activity assessing family functioning (89%).

Valid accelerometer wear was 835.6 ± 76.5 and 734.9.4 ± 62.7 min for adults and children across time points, respectively. Valid accelerometer data on ≥3 days (including 1 weekend day) was available for 82% of adults and 77% of children over the 3 measurement time points. On average across time points, the GPS provided a location for 757.0 ± 126.3 and 541.6 ± 200.3 min for adults and children across time points, respectively.

### Preliminary effectiveness

Levels of MVPA and sedentary behaviour for children and adults are presented in Table 6, subgroup analyses are in Supplementary Tables 1 and 2 and family co-participation in physical activity is available in Supplementary Table 3. Children and adults were either meeting or close to meeting recommended levels of MVPA at baseline, with the exception of FAM children who accumulated notably less MVPA compared to PED and CON children.

**Table 6:** Childrens’ and adults’ mean ± standard deviation daily minutes in moderate-to-vigorous physical activity and sedentary time

	Family			Pedometer			Control			Adjusted difference between groups^**1**^					
										Family vs control		Family vs Pedometer		Pedometer vs Control	
	Baseline (T1)	Change from baseline (T2-T1)	Change from baseline (T3-T1)	Baseline (T1)	Change from baseline (T2-T1)	Change from baseline (T3-T1)	Baseline (T1)	Change from baseline (T2-T1)	Change from baseline (T3-T1)	Mean change (95% CI) T2-T1	Mean change (95% CI) T3-T1	Mean change (95% CI) T2-T1	Mean change (95% CI) T3-T1	Mean change (95% CI) T2-T1)	Mean change (95% CI) T3-T1
**Children**															
N	24	15	15	24	18	15	25	23	22						
MVPA	48.4 ± 15.8	−8.0 ± 13.1	−14.8 ± 17.4	60.5 ± 22.5	−7.3 ± 14.2	−6.4 ± 16.4	54.2 ± 20.4	−4.7 ± 10.3	− 8.4 ± 14.6	−3.1 (−9.9, 3.8)	−3.9 (− 13.7, 5.9)	0.0 (− 8.2, 8.1)	−3.1 (−9.2, 15.4)	− 3.0 (− 10.1, 4.1)	−0.8 (− 10.8, 9.3)
SED	552.3 ± 59.1	−2.6 ± 62.9	−28.6 ± 59.2	469.1 ± 56.5	−1.6 ± 78.1	46.5 ± 52.5	524.6 ± 70.1	− 4.2 ± 56.1	2.4 ± 62.3	7.1 (−18.0, 32.2)	12.3 (− 12.3, 36.8)	8.5 (− 21.2, 38.3)	10.1 (−21.0, 41.2)	−1.5 (− 27.5, 24.5)	2.2 (−23.3, 27.6)
**Adults**															
N	21	16	15	21	17	13	17	19	18						
MVPA	52.0 ± 17.7	2.1 ± 14.8	− 4.6 ± 16.3	52.0 ± 19.2	−12.9 ± 17.7	− 0.9 ± 10.5	47.8 ± 16.3	−7.1 ± 11.3	0.7 ± 17.6	9.4 (0.4, 18.4)	−5.7 (−16.7, 5.3)	15.3 (6.0, 24.5)	1.2 (−11.7, 14.2)	−5.8 (−15.1, 3.3)	− 6.9 (−19.3, 5.5)
SED	647.3 ± 92.6	−17.3 ± 86.5	−49.2 ± 45.9	604.3 ± 70.9	6.8 ± 65.7	19.9 ± 55.1	648.1 ± 55.4	18.4 ± 50.1	− 39.5 ± 68.4	− 17.2 (− 41.3, 7.0)	7.5 (− 17.2, 32.3)	−8.6 (− 33.9, 16.7)	− 6.3 (− 23.2, 35.8)	−8.6 (− 33.0, 15.9)	13.8 (− 14.1, 41.7)

**Note.** ^1^Adjusted for baseline moderate-to-vigorous physical activity or sedentary time, wear time, sex, age. **Abbreviations:** MVPA = moderate-to-vigorous physical activity; SED = sedentary time; T2 = Time 2 assessments 8-weeks post-baseline; T3 = Time 3 assessments 52-weeks post-baseline

In children, there were no notable between-group differences found for minutes in MVPA, time spent sedentary, or co-participation in physical activity with family members. However, a sizeable change of 9.4 (95% CI: 0.4, 18.4) and 15.3 (95% CI: 6.0, 24.5) minutes in MVPA was found for adults in the FAM group compared to those in the PED or CON groups, respectively. Adults in the FAM group also did more activity together compared to the CON and PED groups where there was a change of 11.2 (95% CI: − 2.9, 25.4) and 15.8 (95% CI: 0.5, 31.0) mins, respectively, although in both cases, adult activity was not maintained at 52-weeks. No between-group group differences were found for time spent sedentary in adults.

Exploratory subgroup analyses showed a greater decline in MVPA for FAM girls and FAM children from less deprived areas compared to their counterparts. The latter group also showed a greater increase in sedentary behaviour. In contrast, FAM adults, in particular men, showed a greater increase in MVPA at 8-weeks.

Supplementary Tables 4, 5, 6 display the findings for children and adults for all other outcomes. There were no other notable between-group or subgroup differences found for any other outcome measured at 8- or 52-weeks for children and adults.

### Evaluation of costs

The proportion of families who bought any sports items was materially unaltered throughout the study. Table 7 reports the costs incurred by the family and the intervention cost. The summation of the costs from randomisation to 52-week follow up showed that FAM arm expenditure was on average £157.92 (95% CI: − 154.76, 484.79) more than CON. The majority of this cost difference is accounted for by the cost of the intervention, which is covered by the local authorities. Conversely, CON family expenditure tended to be greater than PED family where an average of £90.50 (95% CI: -£301.30, 104.45) was spent.

**Table 7:** Average costs (95% CI) in pound sterling aggregated at family-level

	Control (*n* = 14)	Family (n = 14)	Pedometer (*n* = 13)
T1	200.9 (131.0, 270.8)	195.1 (110.2, 279.9)	183.1 (118.1, 248.1)
Intervention^+^	N/A	90.0 (84.5, 95.4)	24.6 (19.0, 30.1)
Pedometers	N/A	22.3 (16.9, 27.7)	22.2 (16.6, 27.7)
Other components	N/A	67.7 (−)	2.4 (−)
T2	115.6 (65.3, 165.9)	89.8 (33.9, 145.7)	104.9 (39.9, 169.9)
T3	322.2 (171.4, 473.0)	409.8 (137.6, 681.9)	239.5 (112.0, 367.0)
Total cost	437.8 (275.9, 599.7)	595.7 (307.5, 883.9)	347.3 (216.3, 478.3)
Unadjusted differences^++^	Reference	157.9 (− 154.76, 484.8)	−90.5 (− 301.3, 104.5)
Adjusted differences^+++^	Reference	191.5 (−62.5, 506.3)	−55.7 (−250.0, 143.6)

**Note.** ^+^Intervention costs incurred by local authorities; ^++^ confidence intervals were calculated using 5000 non-parametric bootstrap replicates; ^+++^ The differences were adjusted for cost at baseline. ***Abbreviations*.** *T1* Baseline, *T2* Time 2 assessments 8-weeks post-baseline, *T3* Time 3 assessments 52-weeks post-baseline

### Progression criteria findings

Table 1 shows the findings for each progression criterion, where each was at least partially met.

## Discussion

Our findings showed that it was feasible to deliver and evaluate a family-targeted physical activity promotion intervention with generally high acceptability from participating families. In addition, each of the pre-specified progression criteria were at least partially met (Table 1). However, we only found a favourable indication of effectiveness for adults and not children, that is, a sizeable positive change in MVPA for adults in the FAM group compared to the other groups. The between-group difference found for adults’ minutes in MVPA was not maintained at 52-weeks follow-up and we also found no notable between-group differences for any other outcome measured at either time point.

Family recruitment posed a substantial challenge, and this progression criterion was not met (i.e., recruiting 20 families/month). Our average recruitment rate was ~ 7 families/month (range = 2–15 families/month) despite using a multi-faceted recruitment strategy that targeted adults and children, included a wide range of settings, and direct and indirect recruitment strategies. The recruitment of participants into intervention research has been notoriously difficult [53, 54]. A review of 73 publicly funded trials in the UK (through the National Institute for Health Research) found that only 55% recruited 100% of their target sample size within their pre-agreed timescale and nearly 45% received an extension of some kind [55]. Several studies have reported that the recruitment of families is particularly challenging [13, 56] and we have described specific recruitment challenges we have encountered previously [19]. However, the extent to which under-recruitment occurs in family-based research in unclear. A recent systematic review and Delphi survey investigating effective and resource efficient strategies for recruiting families in physical activity, diet, and obesity prevention research identified 48 eligible studies of which only 31% of studies reported a target sample size [Guagliano JM, Morton KL, Hughes C, van Sluijs EMF, unpublished data]. A subsequent survey showed that only 38% recruited their target sample size over a median (interquartile range) of 12 (7.5–52) weeks. Recruitment periods were extended in 33% studies with a median extension of 20 (8–37.5) weeks [Guagliano JM, Morton KL, Hughes C, van Sluijs EMF, unpublished data]. In terms of recruitment, 94% of adults reported their ethnicity as white. While this figure is reflective of the population of the counties where recruitment occurred [57], the potential effectiveness of this intervention on minority families is unclear. Several studies have acknowledged the underrepresentation of minority groups in trials [58, 59]. Therefore, further research is needed to better establish regarding how to recruit families in family-based research is needed, and in particular, greater consideration should be given to recruiting families of ethnic minority groups. Targeting specific recruitment settings or tailored messaging on recruitment materials are strategies that could be used [59, 60].

An extensive measurement protocol was applied in both the FRESH feasibility [19] and the current study, and it is not possible to disentangle whether the challenges of recruiting families were due to families having a lack of interest in increasing their physical activity, a lack of interest in FRESH in particular, or that the commitment to three rounds of home-based assessment of all family members was a barrier. Families in both the FRESH feasibility [19] and pilot studies indicated that the level of measurement was acceptable to them, but this is likely to be a biased perception of a group of families that has made the commitment to take part in the FRESH study. Further research is needed to identify whether families may not be interested in physical activity promotion per se, or whether the research commitment required poses a barrier. With this in mind, researchers and funders should carefully balance the scientific need for detailed data collection (driven for example by questions around how interventions work, and impacts on important physical health outcomes beyond the target behaviour) with the burden on participants and its impact on recruitment of a representative sample of participants.

Encouragingly, we found evidence of preliminary short-term effectiveness for adults and, in particular, for fathers in the FAM group. Similar interventions with mothers have resulted in positive physical activity promotion [61]. However, the effect on fathers may be noteworthy as evidence indicates that fathers have an independent influence on their children’s health and development [62] and an important influence on children’s physical activity [63–65].

Similar to other family-based physical activity interventions [13, 16, 61], we did not find evidence of preliminary effectiveness for children or for co-participation in physical activity between parents and their children in this study. This may be due to a number of reasons. First, our process evaluation and focus groups revealed that family planning time was not being implemented as intended. In a family-based physical activity intervention that included a similar planning component, the authors found that children’s MVPA significantly increased in the short-term compared to a condition that received education only [66]. Therefore, without implementing the planning component in our study, the step challenges alone may have not been enough to change children’s MVPA. There were also group differences in children’s sex and age, with fewer girls in the PED group and more older children in the FAM group. Observational data reveal that children’s physical activity declines with age [8–10] and girls accumulate less physical activity than boys throughout childhood [28, 67], and girls’ physical activity declines more precipitously than boys with age [68–70]. These differences may have affected preliminary intervention effectiveness on MVPA, but this issue would likely be resolved through randomisation in an adequately powered trial. Lastly, there may have a been a healthy volunteer bias as participants across groups were generally already meeting physical activity recommendations at baseline. In future, excluding families that are sufficiently active could be considered.

Delivery of the FRESH intervention was estimated to cost £90 per family (~£15 per participant), including pedometers for all family members, face-to-face kick-off meeting and personalised follow-up support. The latter accounted for ~ 55% of the costs. These costs could be reduced in future as this part of the intervention delivery had not been automated, but was processed manually by research staff. Further automation of these processes will help reduce delivery costs, and make it more attractive to funding agencies to consider delivering FRESH as part of their portfolio of physical activity interventions, if proven effective. Previous work has estimated the cost of delivering a multi-component school-based physical activity intervention at ~£190/participant [71], and an after-school intervention at £51/participant [72], suggesting that cost of delivering the FRESH intervention is low in comparison. However, little is known about how much local authorities or other delivery agents are willing to pay, and future research should explore this.

### Strengths and limitations

There are several noteworthy strengths of this study which include high retention rates, device-measured physical activity, a measure of family functioning, and a long-term follow-up assessment (i.e., 52-weeks post-baseline). There were also some limitations.

Despite bolstering our recruitment strategy after our feasibility study, we were still unable to recruit the desired number of families into this study; so further optimisation regarding recruitment in family-based research appears prudent. Also, the children and adults that participated in this pilot study were generally sufficiently physically active at baseline, which may have affected the potential of the intervention. Lastly, randomisation did not lead to balanced groups as there were large differences in sex and age among children across groups, where there were noticeably less girls in the PED group and older children in the FAM group. This may have affected our findings for preliminary intervention effectiveness. The randomisation procedure was likely affected by small sample size and the use of a stratified randomisation procedure by county due to funding. There is no indication that this issue would also affect an adequately powered trial; however, stratified randomisation by child sex and/or age could also be considered.

## Conclusion

In conclusion, this study demonstrates feasibility and acceptability of the family-targeted FRESH intervention, as well as satisfying all progression criteria, at least partially. However, we failed to recruit the target sample size and did not find a signal of effectiveness on MVPA particularly long-term or in children. Therefore, further refinements around intervention delivery and recruitment may be required prior to progressing to a full-scale trial.

## Supplementary information

**Additional file 1 Supplementary Table 1.** Childrens’ mean ± standard deviation daily minutes in moderate-to-vigorous physical activity and sedentary time.

**Additional file 2 Supplementary Table 2.** Adults’ mean ± standard deviation daily minutes in moderate-to-vigorous physical activity and sedentary time.

**Additional file 3 Supplementary Table 3.** Mean daily minutes of family co-participation in light, moderate, and vigorous physical activity.

**Additional file 4 Supplementary Table 4.** Secondary outcomes for children.

**Additional file 5 Supplementary Table 5.** Secondary outcomes for adults.

**Additional file 6 Supplementary Table 6.** Family functioning.

## Data Availability

Data for research purposes are available upon request.

## Abbreviations

FRESH: Families Reporting Every Step to Health
GPS: Global positioning system
MVPA: Moderate-to-vigorous physical activity
