# Supplementary material for: A whole family-based physical activity promotion intervention: findings from the families reporting every step to health (FRESH) pilot randomised controlled trial
Source: Int J Behav Nutr Phys Act. 2020 Sep 22;17:120. doi: 10.1186/s12966-020-01025-3 (PMC7510101; doi:10.1186/s12966-020-01025-3)
Supplement: Supplementary file 1 — Additional file 1 Supplementary Table 1. Childrens’ mean ± standard deviation daily minutes in moderate-to-vigorous physical activity and sedentary time. [file 12966_2020_1025_MOESM1_ESM.docx]

| **Supplementary Table 1.** Childrens' mean ± standard deviation daily minutes in moderate-to-vigorous physical activity and sedentary time. | | | | | | | | | |
| --- | --- | --- | --- | --- | --- | --- | --- | --- | --- |
|  | **Family** | | | **Pedometer** | | | **Control** | | |
|  | Baseline  (T1) | Change from baseline (T2-T1) | Change from baseline (T3-T1) | Baseline  (T1) | Change from baseline (T2-T1) | Change from baseline (T3-T1) | Baseline  (T1) | Change from baseline (T2-T1) | Change from baseline (T3-T1) |
| **Boys** |  |  |  |  |  |  |  |  |  |
| N | 11 | 5 | 9 | 17 | 15 | 12 | 14 | 12 | 13 |
| MVPA | 50.3 ± 18.2 | -0.7 ± 11.1 | -12.5 ± 18.3 | 62.8 ± 23.9 | -9.2 ± 14.5 | -7.3 ± 16.9 | 60.2 ± 22.6 | -2.3 ± 10.3 | -6.8 ± 15.6 |
| SED | 569.9 ± 70.0 | -47.5 ± 38.8 | -41.5 ± 67.7 | 469.5 ± 61.5 | -1.6 ± 83.8 | 48.3 ± 53.9 | 520.2 ± 68.8 | -12.5 ± 71.4 | 3.7 ± 67.3 |
| **Girls** |  |  |  |  |  |  |  |  |  |
| N | 13 | 10 | 6 | 4 | 3 | 3 | 11 | 11 | 9 |
| MVPA | 46.8 ± 18.2 | -11.7 ± 13.0 | -18.4 ± 17.1 | 50.9 ± 12.8 | 2.2 ± 9.8 | -2.8 ± 16.9 | 46.5 ± 17.8 | -7.3 ± 10.0 | -10.8 ± 13.5 |
| SED | 537.5 ± 45.2 | 19.9 ± 61.7 | -9.1 ± 41.4 | 467.4 ± 32.6 | -1.3 ± 51.8 | 39.4 ± 56.6 | 530.2 ± 74.7 | 4.9 ± 33.6 | 0.6 ± 63.6 |
| **Index child** |  |  |  |  |  |  |  |  |  |
| N | 11 | 7 | 8 | 12 | 11 | 8 | 14 | 13 | 13 |
| MVPA | 51.6 ± 9.7 | -9.8 ± 11.7 | -13.5 ± 17.4 | 63.0 ± 23.0 | -10.0 ± 14.5 | -8.9 ± 16.3 | 56.2 ± 23.9 | -2.5 ± 10.9 | -5.6 ± 16.0 |
| SED | 537.0 ± 64.9 | -17.4 ± 54.4 | -32.5 ± 54.8 | 470.9 ± 62.0 | 2.1 ± 89.2 | 52.3 ± 43.2 | 532.9 ± 72.3 | -12.5 ± 65.0 | 3.0 ± 43.7 |
| **Additional child** |  |  |  |  |  |  |  |  |  |
| N | 12 | 7 | 6 | 9 | 7 | 7 | 11 | 10 | 9 |
| MVPA | 45.9 ± 20.5 | -4.1 ± 14.3 | -16.2 ± 20.5 | 57.3 ± 22.7 | -3.2 ± 17.3 | -3.6 ± 17.3 | 51.6 ± 15.4 | -7.5 ± 9.2 | -12.4 ± 11.9 |
| SED | 565.9 ± 54.9 | -0.6 ± 68.4 | -24.8 ± 74.4 | 466.6 ± 51.6 | -7.4 ± 63.0 | 39.9 ± 64.5 | 514.0 ± 67.8 | 6.7 ± 42.7 | 1.6 ± 89.4 |
| **Less Deprived** |  |  |  |  |  |  |  |  |  |
| N | 10 | 7 | 3 | 15 | 14 | 12 | 14 | 14 | 13 |
| MVPA | 41.6 ± 9.0 | -12.0 ± 14.2 | -11.4 ± 5.3 | 56.2 ± 23.5 | -5.9 ± 13.4 | -1.9 ± 14.3 | 50.5 ± 17.3 | -5.5 ± 9.8 | -5.5 ± 9.8 |
| SED | 533.7 ± 49.6 | 36.6 ± 51.2 | 19.4 ± 64.4 | 477.2 ± 41.8 | 6.8 ± 61.4 | 43.9 ± 46.8 | 517.5 ± 74.8 | -8.1 ± 63.2 | 3.5 ± 78.4 |
| **More Deprived** |  |  |  |  |  |  |  |  |  |
| **N** | 14 | 8 | 12 | 6 | 4 | 3 | 11 | 9 | 9 |
| MVPA | 53.4 ± 18.0 | -4.5 ± 11.9 | -15.7 ± 19.4 | 71.5 ± 16.6 | -12.5 ± 18.0 | -24.4 ± 12.2 | 58.8 ± 23.8 | -3.4 ± 11.5 | -8.9 ± 18.0 |
| SED | 565.6 ± 63.4 | -36.9 ± 52.8 | -40.6 ± 54.0 | 448.6 ± 84.6 | -30.9 ± 129.4 | 57.0 ± 83.8 | 533.5 ± 66.1 | 2.1 ± 45.6 | 0.8 ± 40.2 |
| **Note.** ^1^Adjusted for baseline moderate-to-vigorous physical activity or sedentary time, wear time, sex, age. **Abbreviations:** MVPA = moderate-to-vigorous physical activity; SED = sedentary time; T2 = Time 2 assessments 8-weeks post-baseline; T3 = Time 3 assessments 52-weeks post-baseline. | | | | | | | | | |
