# Supplementary material for: A whole family-based physical activity promotion intervention: findings from the families reporting every step to health (FRESH) pilot randomised controlled trial
Source: Int J Behav Nutr Phys Act. 2020 Sep 22;17:120. doi: 10.1186/s12966-020-01025-3 (PMC7510101; doi:10.1186/s12966-020-01025-3)
Supplement: Supplementary file 2 — Additional file 2 Supplementary Table 2. Adults’ mean ± standard deviation daily minutes in moderate-to-vigorous physical activity and sedentary time. [file 12966_2020_1025_MOESM2_ESM.docx]

| **Supplementary Table 2.** Adults' mean ± standard deviation daily minutes in moderate-to-vigorous physical activity and sedentary time. | | | | | | | | | |
| --- | --- | --- | --- | --- | --- | --- | --- | --- | --- |
|  | **Family** | | | **Pedometer** | | | **Control** | | |
|  | Baseline  (T1) | Change from baseline (T2-T1) | Change from baseline (T3-T1) | Baseline  (T1) | Change from baseline (T2-T1) | Change from baseline (T3-T1) | Baseline  (T1) | Change from baseline (T2-T1) | Change from baseline (T3-T1) |
| **Men** |  |  |  |  |  |  |  |  |  |
| N | 10 | 7 | 7 | 9 | 7 | 5 | 6 | 8 | 7 |
| MVPA | 55.0 ± 23.1 | 2.3 ± 21.3 | 0.4 ± 11.3 | 61.3 ± 18.5 | -23.5 ± 19.4 | 3.7 ± 11.1 | 42.9 ± 18.4 | -11.8 ± 7.7 | 7.3 ± 17.1 |
| SED | 642.8 ± 108.4 | 2.8 ± 88.61 | -23.4 ± 30.0 | 612.9 ± 60.5 | 1.9 ± 82.1 | -16.1 ± 55.1 | 661.7 ± 44.4 | 14.6 ± 68.6 | -54.7 ± 62.5 |
| **Women** |  |  |  |  |  |  |  |  |  |
| N | 11 | 9 | 8 | 12 | 10 | 8 | 11 | 11 | 11 |
| MVPA | 49.3 ± 11.5 | 2.0 ± 8.4 | -9.0 ± 19.4 | 45.0 ± 17.2 | -5.5 ± 12.5 | -3.8 ± 9.8 | 50.5 ± 15.2 | -3.8 ± 12.6 | -3.5 ± 17.4 |
| SED | 651.3 ± 81.0 | -33.0 ± 86.6 | -71.8 ± 46.9 | 597.8 ± 79.9 | 10.3 ± 56.2 | 42.4 ± 44.5 | 640.7 ± 61.3 | 21.1 ± 34.7 | -29.9 ± 73.0 |
| **Index Parent** |  |  |  |  |  |  |  |  |  |
| N | 12 | 10 | 8 | 13 | 11 | 8 | 13 | 13 | 13 |
| MVPA | 47.7 ± 11.8 | 0.8 ± 14.9 | -6.4 ± 18.3 | 46.0 ± 16.8 | -8.7 ± 15.9 | -3.8 ± 9.8 | 46.2 ± 17.6 | -4.1 ± 11.7 | 1.1 ± 19.5 |
| SED | 653.0 ± 99.6 | -13.5 ± 92.1 | -56.6 ± 44.8 | 591.8 ± 79.8 | 19.1 ± 60.8 | 42.4 ± 44.5 | 640.4 ± 61.2 | 11.8 ± 51.6 | -34.3 ± 67.5 |
| **Additional Parent** |  |  |  |  |  |  |  |  |  |
| N | 9 | 6 | 7 | 8 | 6 | 5 | 4 | 6 | 5 |
| MVPA | 57.8 ± 23.1 | 4.4 ± 15.7 | -2.5 ± 14.9 | 61.9 ± 19.7 | -20.7 ± 19.6 | 3.7 ± 11.1 | 52.9 ± 11.1 | -13.8 ± 7.0 | -0.3 ± 13.3 |
| SED | 639.6 ± 87.7 | -23.7 ± 84.0 | -40.7 ± 49.2 | 624.4 ± 52.1 | -15.6 ± 74.1 | -16.1 ± 55.1 | 673.3 ± 16.6 | 33.6 ± 47.7 | -53.2 ± 76.5 |
| **Less Deprived** |  |  |  |  |  |  |  |  |  |
| N | 6 | 4 | 2 | 13 | 12 | 9 | 10 | 10 | 10 |
| MVPA | 50.4 ± 4.4 | -4.0 ± 14.7 | -7.0 ± 7.4 | 52.4 ± 19.8 | -12.2 ± 13.2 | -3.0 ± 10.2 | 47.3 ± 16.8 | -4.9 ± 12.6 | 3.9 ± 20.1 |
| SED | 547.8 ± 69.5 | 97.5 ± 40.2 | 11.0 ± 2.8 | 616.6 ± 80.6 | 6.5 ± 61.1 | 3.5 ± 53.5 | 622.1 ± 55.9 | 5.3 ± 55.9 | -46.8 ± 86.6 |
| **More Deprived** |  |  |  |  |  |  |  |  |  |
| N | 15 | 12 | 13 | 8 | 5 | 4 | 7 | 9 | 8 |
| MVPA | 48.5 ± 16.7 | 4.2 ± 14.9 | -4.2 ± 17.4 | 51.5 ± 19.4 | -14.6 ± 27.8 | 3.8 ± 11.1 | 48.5 ± 16.7 | -9.7 ± 9.7 | -3.3 ± 14.3 |
| SED | 687.0 ± 67.8 | -55.6 ± 58.0 | -58.5 ± 42.0 | 584.2 ± 50.1 | 7.5 ± 83.8 | 56.9 ± 43.5 | 685.2 ± 27.9 | 32.9 ± 41.0 | -30.5 ± 39.2 |
| **Note.** ^1^Adjusted for baseline moderate-to-vigorous physical activity or sedentary time, wear time, sex, age. **Abbreviations:** MVPA = moderate-to-vigorous physical activity; SED = sedentary time; T2 = Time 2 assessments 8-weeks post-baseline; T3 = Time 3 assessments 52-weeks post-baseline. | | | | | | | | | |
