# Supplementary material for: A whole family-based physical activity promotion intervention: findings from the families reporting every step to health (FRESH) pilot randomised controlled trial
Source: Int J Behav Nutr Phys Act. 2020 Sep 22;17:120. doi: 10.1186/s12966-020-01025-3 (PMC7510101; doi:10.1186/s12966-020-01025-3)
Supplement: Supplementary file 3 — Additional file 3 Supplementary Table 3. Mean daily minutes of family co-participation in light, moderate, and vigorous physical activity. [file 12966_2020_1025_MOESM3_ESM.docx]

| **Supplementary Table 3.** Mean daily minutes of family co-participation in light, moderate, and vigorous physical activity. | | | | | | | | | |
| --- | --- | --- | --- | --- | --- | --- | --- | --- | --- |
|  | **Family** | | | **Pedometer** | | | **Control** | | |
|  | Baseline  (T1) | Change from baseline (T2-T1) | Change from baseline (T3-T1) | Baseline  (T1) | Change from baseline (T2-T1) | Change from baseline (T3-T1) | Baseline  (T1) | Change from baseline (T2-T1) | Change from baseline (T3-T1) |
| **Children** |  |  |  |  |  |  |  |  |  |
| N | 22 | 13 | 16 | 14 | 13 | 12 | 22 | 19 | 20 |
| Child-child LMVPA | 90.4 ± 59.0 | -58.5 ± 67.4 | -38.9 ± 77.0 | 117.1 ± 69.6 | -55.0 ± 110.0 | -75.4 ± 68.0 | 80.2 ± 49.3 | -31.5 ± 68.0 | -28.4 ± 54.3 |
| N | 22 | 19 | 18 | 18 | 19 | 17 | 23 | 23 | 22 |
| Adult-child LMVPA | 46.0 ± 35.7 | -27.5 ± 46.3 | -9.1 ± 39.4 | 40.0 ± 22.5 | -3.4 ± 33.8 | -10.0 ± 36.4 | 55.0 ± 42.4 | -28.2 ± 49.0 | -18.6 ± 53.5 |
| **Adults** |  |  |  |  |  |  |  |  |  |
| N | 14 | 12 | 16 | 16 | 16 | 14 | 8 | 12 | 12 |
| Adult-adult LMVPA | 19.4 ± 14.0 | -1.1 ± 6.1 | -6.7 ± 22.1 | 13.7 ± 10.6 | -1.3 ± 17.6 | -0.7 ± 13.5 | 25.0 ± 19.0 | -10.4 ± 11.5 | -7.8 ± 9.7 |
| N | 21 | 18 | 16 | 18 | 18 | 15 | 19 | 19 | 18 |
| Adult-child LMVPA | 53.4 ± 62.0 | -37.0 ± 77.1 | -29.8 ± 22.8 | 54.9 ± 28.6 | -12.3 ± 30.4 | -29.8 ± 22.8 | 58.6 ± 47.0 | -22.2 ± 57.6 | -14.6 ± 61.9 |
| **Abbreviations:** LMVPA = light, moderate, and vigorous physical activity; T2 = Time 2 assessments 8-weeks post-baseline; T3 = Time 3 assessments 52-weeks post-baseline. | | | | | | | | | |
|  |  |  |  |  |  |  |  |  |  |
