# Supplementary material for: A whole family-based physical activity promotion intervention: findings from the families reporting every step to health (FRESH) pilot randomised controlled trial
Source: Int J Behav Nutr Phys Act. 2020 Sep 22;17:120. doi: 10.1186/s12966-020-01025-3 (PMC7510101; doi:10.1186/s12966-020-01025-3)
Supplement: Supplementary file 4 — Additional file 4 Supplementary Table 4. Secondary outcomes for children. [file 12966_2020_1025_MOESM4_ESM.docx]

| **Supplementary Table 4.** Secondary outcomes for children. | | | | | | | | | | | | |
| --- | --- | --- | --- | --- | --- | --- | --- | --- | --- | --- | --- | --- |
|  | **Family** | | | | **Pedometer** | | | | **Control** | | | |
|  | N | Baseline  (T1) | Change from baseline (T2-T1) | Change from baseline (T3-T1) | N | Baseline  (T1) | Change from baseline (T2-T1) | Change from baseline (T3-T1) | N | Baseline  (T1) | Change from baseline (T2-T1) | Change from baseline (T3-T1) |
| Weight  (Z-score) | 30 | 0.27 ± 0.85 | 0.04 ± 0.18 | 0.11 ± 0.22 | 22 | 0.64 ± 0.86 | 0.00 ± 0.14 | -0.06 ± 0.28 | 29 | 0.01 ± 1.0 | 0.06 ± 0.11 | 0.04 ± 0.27 |
| Waist circumf-erence  (Z-score) | 30 | 0.57 ± 1.33 | 0.14 ± 0.64 | 0.04 ± 0.66 | 22 | 1.17 ± 0.94 | -0.21 ± 0.61 | -0.41 ± 0.57 | 29 | 0.37 ± 1.21 | 0.13 ± 0.71 | -0.01 ± 0.59 |
| Body mass index  (Z-score) | 30 | 0.16 ± 1.09 | 0.01 ± 0.26 | 0.08 ± 0.25 | 22 | 0.29 ± 1.07 | -0.06 ± 0.27 | -0.14 ± 0.36 | 29 | -0.02 ± 1.12 | 0.03 ± 0.15 | -0.01 ± 0.34 |
| Predicted VO_2 max_ | 25 | 41.3 ± 4.1 | -1.0 ± 3.3 | 1.2 ± 4.7 | 17 | 43.8 ± 5.6 | 0.3 ± 4.0 | 1.9 ± 3.1 | 18 | 42.4 ± 5.2 | -0.7 ± 3.5 | 0.4 ± 3.5 |
| Quality of life | 26 | 0.93 ± 0.03 | -0.02 ± 0.08 | -0.07 ± 0.12 | 19 | 0.89 ± 0.11 | 0.01 ± 0.08 | 0.04 ± 0.17 | 22 | 0.92 ± 0.06 | -0.03 ± 0.05 | 0.02 ± 0.08 |
| Weekly step counts | 24 | 50132 ± 17884 | -6786 ± 18413 | -16904 ± 18156 | 21 | 58797 ± 25106 | -4480 ± 23730 | -5363 ± 20625 | 25 | 55708 ± 14350 | -833 ± 11726 | -1818 ± 15274 |
| Weekly television  (mins) | 23 | 609.1 ± 309.2 | -200.9 ± 281.5 | -221.1 ± 423.1 | 22 | 525.0 ± 380.0 | -26.8 ± 290.7 | -130.6 ± 453.2 | 18 | 406.7 ± 212.4 | 63.5 ± 292.0 | -35.3 ± 303.8 |
| Weekly video games  (mins) | 21 | 217.1 ± 347.5 | -91.4 ± 264.0 | -112.9 ± 408.4 | 22 | 278.2 ± 434.8 | -48.6 ± 308.2 | -15.9 ± 273.7 | 18 | 91.2 ± 255.9 | -18.5 ± 158.3 | 87.6 ± 226.8 |
| Weekly computer (mins) | 23 | 469.6 ± 420.9 | -103.0 ± 273.7 | -187.9 ± 363.3 | 22 | 288.2 ± 292.3 | 26.4 ± 265.9 | 32.4 ± 357.6 | 18 | 257.5 ± 316.5 | 12.5 ± 195.7 | 30.8 ± 194.7 |
| Weekly phone  (mins) | 23 | 133.0 ± 280.2 | -93.6 ± 269.9 | -48.3 ± 125.7 | 22 | 78.2 ± 145.9 | 5.0 ± 139.1 | 10.6 ± 166.0 | 19 | 0.5 ± 2.3 | 26.3 ± 47.2 | 24.7 ± 61.3 |
| **Notes.** Values are mean ± standard deviation. **Abbreviations:** T2 = Time 2 assessments 8-weeks post-baseline; T3 = Time 3 assessments 52-weeks post-baseline. | | | | | | | | | | | | |
