# Supplementary material for: A whole family-based physical activity promotion intervention: findings from the families reporting every step to health (FRESH) pilot randomised controlled trial
Source: Int J Behav Nutr Phys Act. 2020 Sep 22;17:120. doi: 10.1186/s12966-020-01025-3 (PMC7510101; doi:10.1186/s12966-020-01025-3)
Supplement: Supplementary file 5 — Additional file 5 Supplementary Table 5. Secondary outcomes for adults. [file 12966_2020_1025_MOESM5_ESM.docx]

| **Supplementary Table 5.** Secondary outcomes for adults. | | | | | | | | | | | | |
| --- | --- | --- | --- | --- | --- | --- | --- | --- | --- | --- | --- | --- |
|  | **Family** | | | | **Pedometer** | | | | **Control** | | | |
|  | N | Baseline  (T1) | Change from baseline (T2-T1) | Change from baseline (T3-T1) | N | Baseline  (T1) | Change from baseline (T2-T1) | Change from baseline (T3-T1) | N | Baseline  (T1) | Change from baseline (T2-T1) | Change from baseline (T3-T1) |
| Weekly step counts | 21 | 56022 ± 15915 | -3415 ± 15866 | -10368 ± 16319 | 21 | 59174 ± 19245 | -12161 ± 18279 | -5259 ± 11781 | 17 | 58287 ± 18438 | -9980 ± 12565 | 705 ± 17782 |
| Weight (kg) | 23 | 81.3 ± 13.8 | -0.1 ± 1.3 | -1.4 ± 2.8 | 21 | 76.5 ± 13.0 | 0.3 ± 1.3 | 0.3 ± 2.3 | 21 | 76.3 ± 15.7 | 0.3 ± 1.1 | 1.4 ± 2.2 |
| Waist circumference (cm) | 23 | 93.4 ± 12.0 | -1.3 ± 5.3 | -2.2 ± 5.5 | 21 | 86.6 ± 12.5 | -1.3 ± 4.4 | -8.4 ± 30.8 | 21 | 86.9 ± 11.3 | 1.4 ± 3.9 | 2.4 ± 4.4 |
| Body mass index | 23 | 27.5 ± 5.0 | 0.1 ± 0.7 | -0.5 ± 1.0 | 21 | 25.6 ± 3.4 | 0.1 ± 0.6 | -2.1 ± 8.7 | 20 | 26.3 ± 5.3 | 0.0 ± 0.4 | 0.5 ± 0.7 |
| Predicted VO_2 max_ | 22 | 33.2 ± 4.7 | 1.2 ± 3.0 | 1.4 ± 1.7 | 21 | 37.0 ± 4.8 | 0.9 ± 3.5 | 1.3 ± 2.8 | 20 | 35.8 ± 6.1 | -0.2 ± 2.8 | 0.3 ± 5.3 |
| Quality of life | 23 | 75.4 ± 14.0 | 2.0 ± 10.3 | 6.5 ± 11.4 | 22 | 82.7 ± 11.1 | -0.2 ± 12.8 | 1.3 ± 7.9 | 19 | 86.5 ± 8.3 | -2.2 ± 11.8 | -2.8 ± 10.0 |
| **Notes.** Values are mean ± standard deviation. **Abbreviations:** T2 = Time 2 assessments 8-weeks post-baseline; T3 = Time 3 assessments 52-weeks post-baseline. | | | | | | | | | | | | |
