# Supplementary material for: A whole family-based physical activity promotion intervention: findings from the families reporting every step to health (FRESH) pilot randomised controlled trial
Source: Int J Behav Nutr Phys Act. 2020 Sep 22;17:120. doi: 10.1186/s12966-020-01025-3 (PMC7510101; doi:10.1186/s12966-020-01025-3)
Supplement: Supplementary file 6 — Additional file 6 Supplementary Table 6. Family functioning. [file 12966_2020_1025_MOESM6_ESM.docx]

| **Supplementary Table 6.** Family functioning. | | | | | | | | | | | | |
| --- | --- | --- | --- | --- | --- | --- | --- | --- | --- | --- | --- | --- |
|  | **Family** | | | | **Pedometer** | | | | **Control** | | | |
|  | N | Baseline  (T1) | Change from baseline (T2-T1) | Change from baseline (T3-T1) | N | Baseline  (T1) | Change from baseline (T2-T1) | Change from baseline (T3-T1) | N | Baseline  (T1) | Change from baseline (T2-T1) | Change from baseline (T3-T1) |
| Mean turns | 6 | 82.5 ± 21.3 | 10.6 ± 6.3 | 13.4 ± 8.7 | 5 | 76.6 ± 9.9 | -12.6 ± 20.9 | 2.4 ± 25.2 | 5 | 82.8 ± 20.7 | 1.5 ± 14.8 | 29.0 ± 22.4 |
| Positive talk/turn | 14 | 0.06 ± 0.06 | -0.01 ± 0.05 | 0 ± 0.03 | 12 | 0.09 ± 0.05 | -0.03 ± 0.03 | -0.03 ± 0.04 | 13 | 0.07 ± 0.04 | -0.01 ± 0.03 | -0.02 ± 0.03 |
| Activity talk/turn | 14 | 0.10 ± 0.08 | -0.02 ± 0.07 | 0.01 ± 0.04 | 12 | 0.06 ± 0.05 | 0.02 ± 0.05 | 0.02 ± 0.06 | 13 | 0.11 ± 0.12 | -0.01 ± 0.03 | -0.06 ± 0.11 |
| **Abbreviations:** T2 = Time 2 assessments 8-weeks post-baseline; T3 = Time 3 assessments 52-weeks post-baseline. | | | | | | | | | | | | |
